# Data correction pre-processing for electronically stored blood culture results: Implications on microbial spectrum and empiric antibiotic therapy

**DOI:** 10.1186/1472-6947-9-27

**Published:** 2009-06-07

**Authors:** Ojan Assadian, Magda Diab-Elschahawi, Athanasios Makristathis, Alexander Blacky, Walter Koller, Klaus-Peter Adlassnig

**Affiliations:** 1 Clinical Institute for Hygiene and Medical Microbiology, Medical University of Vienna, Division of Hospital Hygiene, Waehringer Guertel 18-20, A-1090 Vienna, Austria; 2 Clinical Institute for Hygiene and Medical Microbiology, Medical University of Vienna, Division of Clinical Microbiology, Waehringer Guertel 18-20, A-1090 Vienna, Austria; 3 Section on Medical Expert and Knowledge-Based Systems, Medical University of Vienna, Spitalgasse 23, A-1090 Vienna, Austria; 4 Medexter Healthcare GmbH, Borschkegasse 7/5, A-1090 Vienna, Austria

## Abstract

**Background:**

The outcome of patients with bacteraemia is influenced by the initial selection of adequate antimicrobial therapy. The objective of our study was to clarify the influence of different crude data correction methods on a) microbial spectrum and ranking of pathogens, and b) cumulative antimicrobial susceptibility pattern of blood culture isolates obtained from patients from intensive care units (ICUs) using a computer based tool, MONI.

**Methods:**

Analysis of 13 ICUs over a period of 7 years yielded 1427 microorganisms from positive results. Three different data correction methods were applied. Raw data method (RDM): Data without further correction, including all positive blood culture results. Duplicate-free method (DFM): Correction of raw data for consecutive patient's results yielding same microorganism with similar antibiogram within a two-week period. Contaminant-free method (CFM): Bacteraemia caused by possible contaminants was only assumed as true bloodstream infection, if an organism of the same species was isolated from > 2 sets of blood cultures within 5 days.

**Results:**

Our study demonstrates that different approaches towards raw data correction – none (RDM), duplicate-free (DFM), and a contaminant-free method (CFM) – show different results in analysis of positive blood cultures. Regarding the spectrum of microorganisms, RDM and DFM yielded almost similar results in ranking of microorganisms, whereas using the CFM resulted in a clinically and epidemiologically more plausible spectrum.

**Conclusion:**

For possible skin contaminants, the proportion of microorganisms in terms of number of episodes is most influenced by the CFM, followed by the DFM. However, with exception of fusidic acid for gram-positive organisms, none of the evaluated correction methods would have changed advice for empiric therapy on the selected ICUs.

## Background

Nosocomial infections are estimated to affect 6–12% of hospitalized patients [1]. Of these infections, bacteraemia and fungaemia have the most significant effect on mortality. The outcome of patients with bacteraemia is influenced by the initial selection of adequate antimicrobial therapy [2]. Generally, selecting the antibiotic of choice for the treatment of an infection is a multi-factorial process, which includes site of infection, intrinsic activity of the drug according to microbiological susceptibility testing results, pharmacokinetics of the drug at the site of infection, and potential side effects. However, before considering these factors, the choice of agent is mainly dependant on knowledge of the organisms likely to be involved. Contrarily, in clinical settings, microbiological verification of an infection and susceptibility of the causative pathogen are usually not available at the time of clinical diagnosis of an infection. In order not to endanger the patient, a calculated (empirical) antimicrobial therapy often has to be started without exact knowledge of the causative pathogen and its antimicrobial susceptibility profile. Furthermore,  very little changes occur in the antimicrobial management even at the time when antimicrobial susceptibility results are available to physicians [3].

To assist physicians at an early stage with the empiric antibiotic choice for treating blood stream infections, a close liaison with the clinical microbiologists is important. By regularly monitoring and analyzing blood culture results and by calculating the most frequently isolated microorganisms together with their cumulative susceptibility profiles, the clinical microbiologist can narrow the plausible cause of bacteraemia and susceptibility to antibiotics. Therefore, analysis of occurrence of pathogens and their cumulative susceptibility profiles is widely used in hospitals, and recent attempts on how to standardize these tasks were only developed within the last years [4]. However, applying these definitions manually on large datasets is time consuming, error-prone, and therefore, needs development of expensive software tools.

For blood cultures, there are two concerns that demand correction of crude data before generating statistics on the frequency of occurrence of pathogens and their cumulative antibiotic susceptibility profile. One refers to the fact that in course of treatment and monitoring of the patient, repetitive isolates are obtained, and it is generally believed that by omission of these duplicates the final result will not be biased by multiple cultures of one identical organism during a single infectious episode. The second consideration is the difficulty of interpretation of microbiological test results in distinguishing true episodes of infection from possible contamination of specimens [5]. In clinical practice, merging several other laboratory results together with the clinical aspect of the affected patient makes decision on this subject. Looking at microbiologic data alone often results in overestimation of the real size of the situation, and this is especially true for blood culture results. Several studies have found *Staphylococcus epidermidis *to be the most common microorganism isolated from blood cultures, accounting for 30–60% of all episodes [6-9]. Although these findings were explained partly by the increasing use of intravascular devices, which can serve as portal of entry to the bloodstream, they may be as well due to the retrospective nature of many studies and the lack of criteria for differentiation between contaminated blood cultures and true bacteraemia. Two studies [5,10] could demonstrate that a simplified surveillance definition for nosocomial bloodstream infections based on microbiology data alone yielded comparable result to the Centers for Diseases Control and Prevention's (CDC's) definition for primary bloodstream infection [11] for possible skin contaminant isolates with an agreement rate of 75% [5] and 91% [10], respectively.

Based on these considerations, the objective of our study was to clarify the influence of different crude data correction methods on a) microbial spectrum and ranking of pathogens, and b) cumulative antimicrobial susceptibility pattern of blood culture isolates obtained from patients from intensive care units (ICUs).

## Methods

The Vienna General Hospital is a 2,140 beds tertiary care referral institution that serves as the teaching facility of the Medical University of Vienna. The hospital cares for about 90,000 inpatients annually. Daily, more than 500 patient samples are sent to the Division of Clinical Microbiology for further processing. After analyzing samples, a clinical microbiologist verifies the results, and findings are downloaded to the hospital's central laboratory database, stored and then forwarded to the sample sender. Simultaneously, the results are downloaded to the MONI (Monitoring of Nosocomial Infections) database, which has a relational, Oracle-based architecture located on a separate server. MONI is a database and surveillance system designed to monitor and detect nosocomial infections [12-15]. Currently, the system offers three categories of applications: a) administration tools, which enable to control database settings and adjusting database parameters; b) a database query tool (FlexScan), which allows to extract records in accordance with manually selected restrictions (for example sender, microorganism, sample, antibiotic resistance profile), or to conduct automatic queries based on knowledge-based rules, resulting in stratified standard analysis of proportions of microorganisms and cumulative antimicrobial susceptibility profiles; c) an automatic surveillance tools using rules for monitoring of alert organisms (e.g. methicillin-resistant *Staphylococcus aureus*) and antibiotic resistance profiles (MONI/ALERT), monitoring of cross infections (MONI/CROSS), and monitoring of frequencies and trends (MONI/TREND). The hospital's infection control team analyzes the intelligent alarms generated by MONI, and reacts to them by initiating necessary countermeasures.

No ethical approval was needed for this study, as data acquisition followed Austrian federal law on data safety, and data pre-processing and analysing occurred anonymously.

### Data acquisition and processing

Data acquisition was performed using the system's database query tool FlexScan. Since preliminary reports of blood culture results can introduce considerable redundancy into a database of microbiology results, only the final reports from each sample are downloaded to the MONI database. For purpose of this study, three separate queries were conducted, one using the programs duplicate result filter (duplicate-free method – DFM), the other without any data correction (raw data method – RDM). For the purpose of evaluating a new data correction rule, a third query was performed using definitions published by Yokoe et al. [10] (contaminant-free method – CFM).

Following the selection of a time period (1 January 1998 – 31 December 2004), a sampling material (blood culture), and the wards under study (13 ICUs: 7 surgical, 5 medical, and 1 neuro-surgical), the program was started. After performing the queries, results were stored in a dynamic comma separated (*.csv) file, and analyzed for more detail using a standard spreadsheet application (MS Excel 2000, Microsoft Corporation, Redmond, Virginia). Data from each patient included the following variables: patient identification number, family name, first name, date of birth, patient's age at sample collection, protocol number of the blood culture report, date of sampling, sender's department and ward, sampling material (blood culture), identified organism, and antimicrobial susceptibility pattern in terms of resistant, susceptible, or intermediate susceptible.

### Definition of data correction rules

#### Raw data method (RDM)

Raw data were defined as data acquired by MONI without any further correction. Hence, they included all positive blood culture results obtained from patients of 13 ICUs from 1 January 1998 – 31 December 2004 without any further manipulation.

#### Duplicate-free method (DFM)

**Figure 1:**
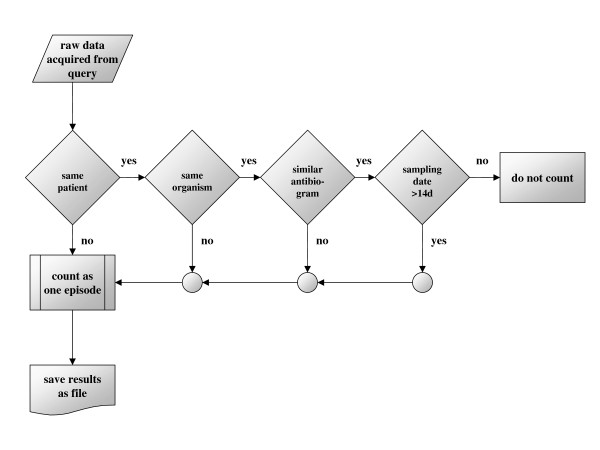
**Flowchart of the "if/then" rules of duplicate-free method (DFM)**.

(figure 1) The duplicate-free method was defined as correction of raw data by elimination of duplicate results. A duplicate result was defined as any result presenting the same microorganism (genus and species) from the same material (in this study blood culture) with a similar antimicrobial susceptibility pattern (at least 85% similarity) obtained from the same patient within a two-week period starting from the last positive culture with the identical organism. Intermediate susceptibility results were also considered as resistant results. The accepted differences of not more than 15% in antibiotic susceptibility was related most to the clinical practice of testing additional antibiotics or to omit re-testing of previously tested compounds. Hence, in most cases the difference pertained more to missing or additionally tested compounds. Only the first result from a patient – representing one episode of bacteraemia – was enrolled for further analysis.

#### Contaminant-free method (CFM)

**Figure 2:**
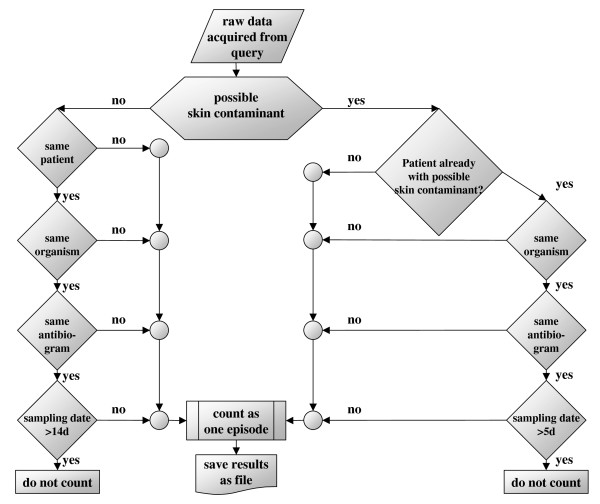
**Flowchart of the "if/then" rules of contaminant-free method (CFM)**.

(figure 2) Considering the problem of contamination with skin organisms, we applied the definitions published earlier [10]. Possible skin contaminants were defined as organisms, which are part of the normal skin flora, including coagulase-negative staphylococci, *Corynebacterium species*, alpha- or non-hemolytic streptococci [5], *Bacillus species*, *Propionibacterium acnes*, micrococci, and *Neisseria species *other than *N. gonorrhoeae *and *N. meningitidis*. All coagulase-negative staphylococci were differentiated on the species level, and not on the genus level alone. Other bacteria and fungi were regarded as obligate pathogens (e.g. *Salmonella typhi*, *Staphylococcus aureus*) and therefore always considered as true cause of bacteraemia, whereby only the first isolate within a 14-day period was counted as one episode. Bacteraemia caused by a possible skin contaminant organism was assumed as true if an organism of the same species with a similar antimicrobial susceptibility pattern (at least 85% similarity) was isolated from two or more sets of blood cultures obtained from the same patient within 5 days starting from the last positive culture with the identical organism. In this case, this was counted as a single episode of bacteraemia. If only one isolate was recorded within this period, the blood culture was regarded to be contaminated and was excluded from further analysis.

### Calculation and interpretation of antimicrobial susceptibility pattern

Because a gram stain is usually the first available information which can be provided to a physician, susceptibility results calculated by each method were summarized for organisms grouped according to their gram stain result. Based on empirical considerations, we excluded an antimicrobial substance as possible choice for the calculated antibiotic treatment of a bacteraemia, if its resistance reached ≥ 10% against a given microorganism. For all isolates, antimicrobial susceptibility testing was performed by Kirby-Bauer disk-diffusion test according to the Clinical and Laboratory Standards Institute (CLSI, before 2005 known as the National Committee for Clinical Laboratory Standards, NCCLS) guidelines [16], if appropriate. Results of cumulative antimicrobial susceptibility were presented as percentage of resistance against a tested antimicrobial substance.

### Statistical analysis

Differences of proportions between RDM, DFM and CFM were calculated by applying the χ^2 ^test, which gives the probability that an observed difference between two means or proportions is caused by chance. A P-value of less than 0.05 was considered significant. Calculations were performed using Epi Info 2000 v 1.1.2a (Centers for Disease Control and Prevention, Atlanta, GA 30333, USA).

## Results

### Microbial spectrum and ranking of microorganisms

Analysis from 13 ICUs (01/1998 – 12/2004) yielded 1427 microorganisms from positive blood culture results. The spectrum of microorganisms, calculated by RDM, DFM, and CFM, respectively, is shown in Table 1. By means of all data correction methods, *Staphylococcus epidermidis *and *Staphylococcus aureus *were the most common organisms. However, regarding their proportion expressed as percentage of all episodes, there are significant differences. Table 2 and Table 3 show the difference of proportions obtained by the different data correction methods as well as the results of significance testing for possible skin contaminants and obligate pathogens, respectively. Although definitions for DFM and CFM did not differ for obligate pathogens, due to the different size of denominators statistically significant differences with respect to proportion of episodes could be observed. While there were differences in proportions for all microorganisms, no statistically significant difference was found for RDM vs. DFM.

**Table 1:** Comparison of the spectrum and ranking of microorganisms identified from blood cultures in 13 ICUs (1998–2004), analyzed by different data pre-processing methods.

**RDM**^a^				**DFM**^b^				**CFM**^c^			
Rank	Microorganism	n	%	Rank	Microorganism	n	%	Rank	Microorganism	n	%
		
1	*Staphylococcus epidermidis*	448	31.4%	1	*Staphylococcus epidermidis*	305	32.1%	1	*Staphylococcus aureus*	123	20.6%
2	*Staphylococcus aureus*	198	13.9%	2	*Staphylococcus aureus*	123	12.9%	2	*Staphylococcus epidermidis*	80	13.4%
3	*Candida albicans*	96	6.7%	3	*Pseudomonas aeruginosa*	58	6.1%	3	*Pseudomonas aeruginosa*	58	9.7%
4	*Pseudomonas aeruginosa*	89	6.2%	4	*Candida albicans*	56	5.9%	4	*Candida albicans*	56	9.4%
5	*Escherichia coli*	71	5.0%	5	*Escherichia coli*	41	4.3%	5	*Escherichia coli*	41	6.9%
6	*Enterobacter cloacae*	63	4.4%	6	*Enterobacter cloacae*	36	3.8%	6	*Enterobacter cloacae*	36	6.0%
7	*Enterococcus faecalis*	45	3.2%	7	*Enterococcus faecalis*	29	3.0%	7	*Enterococcus faecalis*	29	4.8%
8	*Enterococcus faecium*	34	2.4%	8	*Enterococcus faecium*	27	2.8%	8	*Enterococcus faecium*	27	4.5%
9	*Acinetobacter baumannii*	26	1.8%	9	*Klebsiella pneumoniae*	16	1.7%	9	*Klebsiella pneumoniae*	16	2.7%
10	*Staphylococcus haemolyticus*	24	1.7%	10	*Staphylococcus haemolyticus*	15	1.6%	10	*Acinetobacter baumannii*	13	2.2%
	others	333	23.3%		Others	245	25.8%		others	119	19.9%
											
	Total	1427	100.0		Total	951	100.0		total	598	100.0

^a ^RDM = raw data method; ^b ^DFM = duplicate-free method; ^c ^CFM = contaminant-free method.

**Table 2:** Difference of proportions according to applied data correction method for possible skin contaminants.

	RDM vs. DFM			RDM vs. CFM			DFM vs. CFM		
	% difference	χ^2^	p-value	% difference	χ^2^	p-value	% difference	χ^2^	p-value

*S. epidermidis*	0.7%	0.12	0.728	18.0%	70.96	< 0.0001*	18.7%	68.69	< 0.001*
*S. haemolyticus*	0.1%	0.04	0.844	0.6%	1.33	0.249	0.7%	0.90	0.342
*Corynebacterium spp*.	0.2%	0.14	0.705	1.1%	2.26	0.132	1.3%	2.93	0.087

χ^2 ^= Chi-square test; A p-value less than 0.05 (*) is considered to indicate a statistically significant difference.

**Table 3:** Difference of proportions according to applied data correction method for obligate pathogens.

	RDM vs. DFM			RDM vs. CFM			DFM vs. CFM		
	% difference	χ^2^	p-value	% difference	χ^2^	p-value	% difference	χ^2^	p-value

*S. aureus*	1.0%	0.43	0.510	6.7%	14.15	0.0002*	7.7%	16.02	< 0.001*
*C. albicans*	0.8%	0.67	0.413	2.7%	4.22	0.039*	3.5%	6.61	0.010*
*P. aeruginosa*	0.1%	0.02	0.881	3.5%	7.50	0.006*	3.6%	6.93	0.008*

χ^2 ^= Chi-square test; A p-value less than 0.05 (*) is considered to indicate a statistically significant difference.

Among the 333 microorganisms representing 23.3% of all positive blood cultures summarized as "others" in Table 1, *Staphylococcus spp*. (other than *S. epidermidis*, *S. aureus *and *S. haemolyticus*) (68; 4.8%), *Corynebacterium spp*. (39; 2.7%), *Klebsiella spp*. (32; 2.2%), viridans group streptococci (27; 1.9%), *Candida spp*. (other than *C. albicans*) (24; 1.7%), *Propionibacterium spp*. (20; 1.4%), *Streptococcus pneumoniae *(18; 1.3%), *Citrobacter spp*. (10; 0.7%), *Enterobacter spp*. (other than *E. cloacae*) (10; 0.7%), *Serratia marcescens *(9; 0.6%), *Acinetobacter spp*. (other than *A. baumanii*) (7; 0.5%), *Stenotrophomonas maltophilia *(7; 0.5%), *Bacteroides spp*. (6; 0.4%), *Enterococcus spp*. (other than *E. faecalis *and *E. faecium*) (6; 0.4%), *Ralstonia pickettii *(6; 0.4%), *Burkholderia cepacia *(4; 0.3%), *Haemophilus spp*. (4; 0.3%), *Peptostreptococcus anaerobius *(4; 0.3%), *Alcaligenes xylosoxidans *(3; 0.2%), *Micrococcus spp*. (3; 0.2%), *Morganella morganii *(3; 0.2%), *Proteus mirabilis *(3; 0.2%), *Aerococcus viridans *(2), *Clostridium spp*. (2), *Flavimonas oryzihabitans *(2), group B streptococci (2), *Actinomyces meyeri *(1), *Bifidobacterium sp*. (1), *Campylobacter jejuni *(1), *Cryptococcus neoformans *(1), *Gemella sp*. (1), *Kocuria sp*. (1), *Lactobacillus casei *(1), *Listeria monocytogenes *(1), *Prevotella denticola *(1), *Pseudomonas alcaligenes *(1), group F (1), and group G streptococci (1).

#### Results of antimicrobial susceptibility testing and appropriateness of antibiotics for empirical therapy

**Figure 3:**
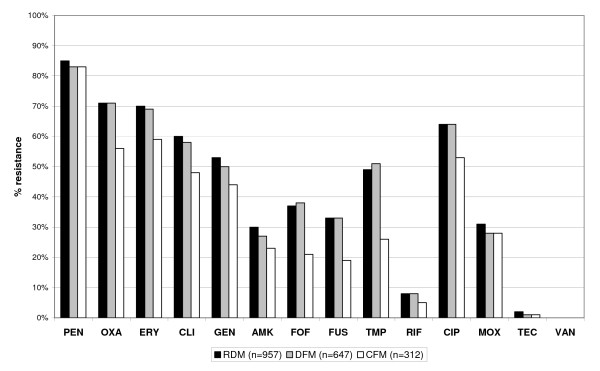
**Cumulative antimicrobial resistance profile of gram positive organisms**. PEN = penicillin; OXA = oxacillin; ERY = erythromycin; CLI = clindamycin; GEN = gentamicin; AMK = amikacin; FOF = fosfomycin; FUS = fusidic acid; TMP = trimethoprim; RIF = rifampicin; CIP = ciprofloxacin; MOX = moxifloxaciln; TEC = teicoplanin; VAN = vancomycin.

**Figure 4:**
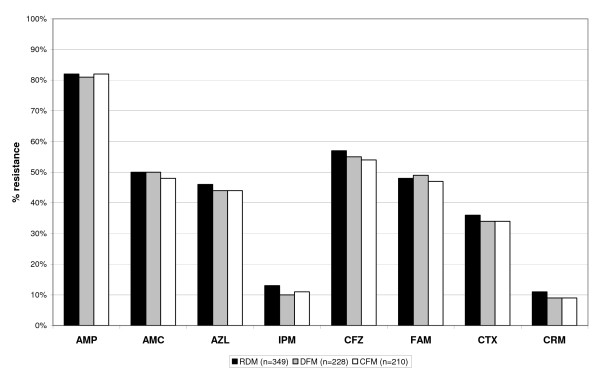
**Cumulative antimicrobial resistance profile of gram negative organisms**. AMP = ampicillin; AMC = amoxicillin-clavulanic acid; AZL = azlocillin; IPM = imipenem; CFZ = cefazolin; FAM = cefamandole; CTX = cefotaxime; CRM = cefpirom.

Figure 3 shows differences in the antimicrobial resistance profile of gram-positive, Figure 4 of gram- negative organisms. Considering an empirical cut-off level of ≥ 20% of resistant organisms to an antibiotic as limit for appropriateness of this agent for empirical therapy of infection caused by gram-negative organisms, no data correction method would have yielded a different recommendation for empirical therapy. However, for gram-positive organisms, only the application of the CFM changed significantly the advice for empiric use of fusidic acid (RDM: 33%, DFM: 33%, and CFM: 19% resistance; P = 0.024).

## Discussion

For small hospitals, a manually conducted review and analysis of microbiological data is achievable, but limited by the small numbers of isolates and therefore ensuing a decreased reliability of results. At large institutions, there is often an enormous pool of microbiological data, yielding more accurate estimations on the prevalence of pathogens, but a manually conducted review is unattainable. A large volume of clinical data demands the use of computer systems, which analyze data in accordance with programmed "if/then" rules derived from models used by medical experts. Naturally, direct transfer of human medical decision-making behaviour to "if/then" rules for artificial intelligence-based computer programs is not always possible, because most of the necessary information is not easily available in electronic form (for example patient temperature, abdominal discomfort, patient "looks" ill). Also, because of the heterogeneity of used laboratory and medical information systems even within the same hospital, merging of information obtained from different sources is often futile. Therefore, the CFM, originally designed as rapid method for surveillance of cases of infection, is an interesting method, although its use strongly depends on the availability of computer systems, since complex "if/then" rules for data correction of large datasets are highly time and concentration consuming and consecutive human errors never can be ruled out.

However, because of the requirement of at least two sets of positive blood cultures within 5 days, the prevalence of true bacteraemia caused by possible skin contaminants may be underestimated by the CFM if the practice of obtaining only one set of blood cultures is common. This situation usually does not arise at ICUs, since in this setting often more than one set of blood culture is drawn on consecutive days. The rationale for this practice is monitoring for success of therapy, and not to miss additional infections by multiple resistant pathogens during antimicrobial therapy.

On the other hand, the CFM enables to study those microorganisms classified as "true" pathogens, and hence being clinically and epidemiologically more relevant. Also, this method gives better estimation of the proportion of the organism isolated from blood cultures. Our study showed a percentage of 13.4% of *Staphylococcus epidermidis *using CFM, which is a very plausible estimation on the real proportion of infections caused by this organism according to previous results of prospective studies on catheter-related infections at ICUs [17]. Regarding susceptibility pattern and implications on empiric antibiotic therapy, the CFM showed only a difference to DFM or RDM for fusidic acid. With regard to the associations of empiric therapy and antimicrobial susceptibilities, the difference for the CFM method for fusidic acid, while an interesting observation, is not relevant to the topic of the study question.

## Conclusion

Our study demonstrates that different approaches towards raw data correction – none (RDM), duplicate-free (DFM), and a contaminant-free method (CFM) – show different results in analysis of positive blood cultures. Regarding the spectrum of microorganisms, RDM and DFM yielded almost similar results in ranking of microorganisms, whereas using the CFM resulted in a clinically and epidemiologically more plausible spectrum. For possible skin contaminants, the proportion of microorganisms in terms of number of episodes is most influenced by the CFM, followed by the DFM. However, with exception of fusidic acid for gram-positive organisms, none of the evaluated correction methods would have changed advice for empiric therapy on the selected ICUs.

## Competing interests

The authors have no competing, financial or other conflict of interest to declare in relation to this manuscript and declare no financial or other relationships leading to a conflict of interest. KPA is co-owner of Medexter Healthcare GmbH, Borschkegasse 7/5, A-1090 Vienna, Austria, a company specialized in development of medical expert systems in medicine. KPA has no financial or other competing conflict of interest related to results or conclusions related to the presented work.

## Authors' contributions

OA had the idea for the study and planned and conducted the experiments, as well drafted and wrote the manuscript, analyzed and interpreted the data. He also conducted the statistical analysis of the results. MDE participated in the technical design of the study and performed re-testing of the calculations, analyzed and interpreted the data. AM helped drafting the material and method section of the paper, particularly on microbiological matters. AB helped to draft the manuscript, and re-analyzed and interpreted the data. WK and KPA advised in design and coordination of the study and provided the technical background for collecting and mining data. All authors have been involved in drafting the manuscript or revising it critically for important intellectual content and have read and approved the final manuscript.

## Pre-publication history

The pre-publication history for this paper can be accessed here:

http://www.biomedcentral.com/1472-6947/9/27/prepub

## References

[B1] Ruden H Gastmeier P Daschner FD Schumacher M Nosocomial and community-acquired infections in Germany. Summary of the results of the First National Prevalence Study (NIDEP) Infection 1997 25 199 202 10.1007/BF01713142 9266256

[B2] Byl B Clevenbergh P Jacobs F Struelens MJ Zech F Kentos A Thys JP Impact of infectious diseases specialists and microbiological data on the appropriateness of antimicrobial therapy for bacteremia Clin Infect Dis 1999 29 60 66 10.1086/520182 10433566

[B3] Munson EL Diekema DJ Beekmann SE Chapin KC Doern GV Detection and treatment of bloodstream infection: laboratory reporting and antimicrobial management J Clin Microbiol 2003 41 495 497 12517905 10.1128/JCM.41.1.495-497.2003 PMC149611

[B4] National Committee on Clinical Laboratory Standards Analysis and Presentation of Cumulative Antimicrobial Susceptibility Test Data; Approved Guideline NCCLS document M39-A 2002 NCCLS, 940 West Valley Road, Suite 1400, Wayne, Pensilvania 19087-1898, USA ISBN 1-56238-422-8

[B5] Richter SS Beekmann SE Croco JL Diekema DJ Koontz FP Pfaller MA Doern GV Minimizing the workup of blood culture contaminants: implementation and evaluation of a laboratory-based algorithm J Clin Microbiol 2002 40 2437 2444 12089259 10.1128/JCM.40.7.2437-2444.2002 PMC120579

[B6] Spencer RC Epidemiology of infection in ICUs Intensive Care Med 1994 20 2 6 10.1007/BF01713975 7699152

[B7] Spencer RC Predominant pathogens found in European prevalence of infection in intensive care study Eur J Clin Microbiol Infect Dis 1996 15 281 285 10.1007/BF01695658 8781877

[B8] Pittet D Tarara D Wenzel RR Nosocomial bloodstream infection in critically ill patients JAMA 1994 271 1598 1601 10.1001/jama.271.20.1598 8182812

[B9] Rello J Ricart M Mirelis B Quintana E Gurgui M Net A Prats G Nosocomial bacteremia in a medical-surgical intensive care unit: epidemiology, characteristics and factors influencing mortality in 111 episodes Intensive Care Med 1994 20 94 98 10.1007/BF01707661 8201105

[B10] Yokoe DS Anderson J Chambers R Connors M Finberg R Hopkins C Lichtenberg D Marino S McLaughlin D O'Rourke E Samore M Sands K Strymish J Tamplin E Vallonde N Platt R Simplified surveillance for nosocomial bloodstream infections Infect Control Hosp Epidemiol 1998 19 657 660 9778164 10.1086/647894

[B11] Garner JS Jarvis WR Emori TG Horan TC Hughes JM CDC definitions for nosocomial infections, 1988 Am J Infect Control 1988 16 128 140 10.1016/0196-6553(88)90053-3 2841893

[B12] Chizzali-Bonfadin C Adlassnig K-P Koller W Greens RA, Peterson HE, Protti DJ An intelligent database and monitoring system for surveillance of nosocomial infections Proceedings of the 8th World Congress on medical informatics (MEDINFO 95): Healthcare computing and communications, Edmonton, Canada 1995 1684 8591544

[B13] Assadian O Adlassnig K-P Rappelsberger A Koller W Adlassnig K-P MONI – An Intelligent Infection Surveillance Software Package Intelligent Systems in Patient Care 2001 Österreichische Computer Gesellschaft, Vienna, Austria 49 56

[B14] Fabini B Monitoring of infectious risk situations and nosocomial infections in the hospital Thesis at the Technical University Vienna, Faculty of technical-natural sciences, Vienna, Austria 2001

[B15] Heisz H Praktisch orientierte Konzepte der Inferenz mit fuzzy-Regeln auf Grundlage des nosokomialen Diagnosesystems MONI-IV Thesis at the Technical University Vienna, Faculty of Informatics, Vienna, Austria 2004

[B16] National Committee on Clinical Laboratory Standards Approved Guideline NCCLS document M100-S8 (1998) NCCLS, 940 West Valley Road, Suite 1400, Wayne, Pensilvania 19087-1898, USA

[B17] Herwaldt LA Geiss M Kao C Pfaller MA The positive predictive value of isolating coagulase-negative staphylococci from blood cultures Clin Infect Dis 1996 22 14 20 8824959 10.1093/clinids/22.1.14

